# Benzyl *N*′-(1*H*-indol-3-ylmethyl­idene)­hydrazinecarbodithio­ate

**DOI:** 10.1107/S160053680803198X

**Published:** 2008-10-11

**Authors:** Hamid Khaledi, Hapipah Mohd Ali, Seik Weng Ng

**Affiliations:** a Department of Chemistry, University of Malaya, 50603 Kuala Lumpur, Malaysia

## Abstract

The C_10_H_8_N_3_S_2_ portion of the title mol­ecule, C_17_H_15_N_3_S_3_, is nearly planar (r.m.s. deviation 0.05 Å); this unit and the phenyl ring subtend an angle of 114.5 (2)° at the methyl­ene C atom.

## Related literature

For other Schiff bases derived by condensing *S*-benzyl hydrazinecarbodithio­ate with either aromatic aldehydes or ketones, see: Ali *et al.* (2004 ▶); Chan *et al.* (2003 ▶); Fun *et al.* (1995 ▶); How *et al.* (2007*a* ▶,*b* ▶,*c* ▶); Khoo *et al.* (2005 ▶); Qiu & Luo (2007 ▶); Roy *et al.* (2007 ▶); Tarafder *et al.* (2002 ▶); Xu *et al.* (1991 ▶); Zhang *et al.* (2004 ▶).
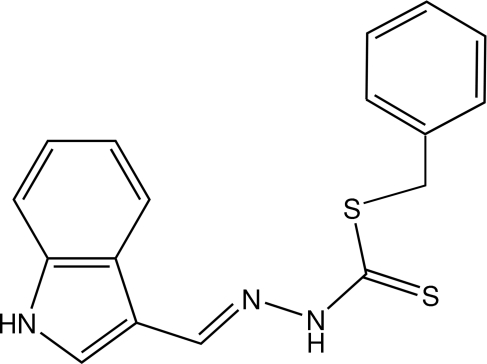


## Experimental

### 

#### Crystal data

C_17_H_15_N_3_S_2_
*M* *_r_* = 325.44
Monoclinic, 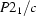

*a* = 15.4936 (7) Å
*b* = 9.8114 (4) Å
*c* = 10.2531 (4) Å
β = 98.432 (3)°
*V* = 1541.8 (1) Å^3^
*Z* = 4
Mo *K*α radiation
μ = 0.34 mm^−1^
*T* = 100 (2) K
0.25 × 0.10 × 0.03 mm

#### Data collection

Bruker SMART APEX diffractometer
Absorption correction: multi-scan (*SADABS*; Sheldrick, 1996 ▶) *T*_min_ = 0.919, *T*_max_ = 0.990
8531 measured reflections
3383 independent reflections
2323 reflections with *I* > 2σ(*I*)
*R*_int_ = 0.058

#### Refinement

*R*[*F* ^2^ > 2σ(*F* ^2^)] = 0.049
*wR*(*F* ^2^) = 0.118
*S* = 1.01
3383 reflections
199 parameters
H-atom parameters constrained
Δρ_max_ = 0.48 e Å^−3^
Δρ_min_ = −0.33 e Å^−3^

### 

Data collection: *APEX2* (Bruker, 2007 ▶); cell refinement: *APEX2*; data reduction: *SAINT* (Bruker, 2007 ▶); program(s) used to solve structure: *SHELXS97* (Sheldrick, 2008 ▶); program(s) used to refine structure: *SHELXL97* (Sheldrick, 2008 ▶); molecular graphics: *X-SEED* (Barbour, 2001 ▶); software used to prepare material for publication: *publCIF* (Westrip, 2008 ▶).

## Supplementary Material

Crystal structure: contains datablocks global, I. DOI: 10.1107/S160053680803198X/tk2309sup1.cif

Structure factors: contains datablocks I. DOI: 10.1107/S160053680803198X/tk2309Isup2.hkl

Additional supplementary materials:  crystallographic information; 3D view; checkCIF report

## Figures and Tables

**Fig. 1.**
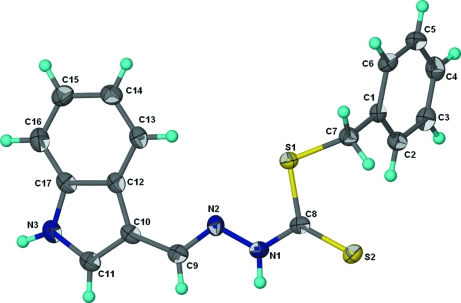
Thermal ellipsoid plot (Barbour, 2001) of C17H15N2S3 at the 70% probability level. Hydrogen atoms are drawn as spheres of arbitrary radius.

## supplementary crystallographic information

### Comment

The structure of (I), Fig. 1, shows bond distances for N1—N2 and N2—C9 of 1.382 (3) and 1.287 (3) Å, respectively, confirming the assignment shown in the Scheme. The molecule is bent about the methylene-C7 atom so that the residues on either side are approximately orthogonal. The amino groups do not form any hydrogen bonds.

### Experimental

Indole-3-carbaldehyde (0.37 g, 2.5 mmol) and *S*-benzyl dithiocarbazate (0.50 g, 2.5 mmol) were heated in methanol (40 ml) for 3 h. The solution was set aside for the formation of yellow crystals.

### Refinement

Hydrogen atoms were placed at calculated positions (C—H 0.95–0.99, N—H 0.88 Å) and were treated as riding on their parent carbon atoms, with *U*(H) set to 1.2 times *U*_eq_(C,N).

### Crystal data

**Table:**

C_17_H_15_N_3_S_2_	*F*(000) = 680
*M**_r_* = 325.44	*D*_x_ = 1.402 Mg m^−^^3^
Monoclinic, *P*2_1_/*c*	Mo *K*α radiation, λ = 0.71073 Å
Hall symbol: -P 2ybc	Cell parameters from 1305 reflections
*a* = 15.4936 (7) Å	θ = 2.5–23.2°
*b* = 9.8114 (4) Å	µ = 0.34 mm^−^^1^
*c* = 10.2531 (4) Å	*T* = 100 K
β = 98.432 (3)°	Prism, light yellow
*V* = 1541.8 (1) Å^3^	0.25 × 0.10 × 0.03 mm
*Z* = 4	

### Data collection

**Table:**

Bruker SMART APEX diffractometer	3383 independent reflections
Radiation source: fine-focus sealed tube	2323 reflections with *I* > 2σ(*I*)
graphite	*R*_int_ = 0.058
ω scans	θ_max_ = 27°, θ_min_ = 1.3°
Absorption correction: multi-scan (SADABS; Sheldrick, 1996)	*h* = −20→20
*T*_min_ = 0.919, *T*_max_ = 0.990	*k* = −12→12
8531 measured reflections	*l* = −13→8

### Refinement

**Table:**

Refinement on *F*^2^	Primary atom site location: structure-invariant direct methods
Least-squares matrix: full	Secondary atom site location: difference Fourier map
*R*[*F*^2^ > 2σ(*F*^2^)] = 0.049	Hydrogen site location: inferred from neighbouring sites
*wR*(*F*^2^) = 0.118	H-atom parameters constrained
*S* = 1.01	*w* = 1/[σ^2^(*F*_o_^2^) + (0.0391*P*)^2^ + 1.1115*P*] where *P* = (*F*_o_^2^ + 2*F*_c_^2^)/3
3383 reflections	(Δ/σ)_max_ = 0.001
199 parameters	Δρ_max_ = 0.48 e Å^−^^3^
0 restraints	Δρ_min_ = −0.33 e Å^−^^3^

### Fractional atomic coordinates and isotropic or equivalent isotropic displacement parameters (Å^2^)

**Table:**

	*x*	*y*	*z*	*U*_iso_*/*U*_eq_	
S1	0.75015 (5)	0.74458 (7)	0.47255 (7)	0.02090 (18)	
S2	0.87340 (5)	0.53986 (7)	0.36891 (7)	0.02198 (19)	
N1	0.70350 (15)	0.5227 (2)	0.3457 (2)	0.0217 (5)	
H1	0.7086	0.4440	0.3063	0.026*	
N2	0.62145 (15)	0.5704 (2)	0.3596 (2)	0.0212 (5)	
N3	0.32371 (16)	0.5105 (2)	0.2983 (2)	0.0253 (6)	
H3	0.2706	0.4795	0.2732	0.030*	
C1	0.87962 (17)	0.9360 (3)	0.4432 (3)	0.0188 (6)	
C2	0.89308 (18)	0.9185 (3)	0.3115 (3)	0.0216 (6)	
H2	0.8855	0.8312	0.2714	0.026*	
C3	0.91737 (18)	1.0283 (3)	0.2403 (3)	0.0242 (7)	
H3a	0.9272	1.0156	0.1518	0.029*	
C4	0.92738 (18)	1.1564 (3)	0.2970 (3)	0.0259 (7)	
H4	0.9436	1.2316	0.2475	0.031*	
C5	0.91353 (19)	1.1742 (3)	0.4261 (3)	0.0245 (7)	
H5	0.9202	1.2619	0.4654	0.029*	
C6	0.89010 (18)	1.0647 (3)	0.4983 (3)	0.0212 (6)	
H6	0.8811	1.0780	0.5871	0.025*	
C7	0.85739 (18)	0.8174 (3)	0.5249 (3)	0.0210 (6)	
H7A	0.8609	0.8475	0.6176	0.025*	
H7B	0.9018	0.7453	0.5220	0.025*	
C8	0.77530 (18)	0.5938 (3)	0.3909 (3)	0.0191 (6)	
C9	0.55868 (18)	0.4859 (3)	0.3251 (3)	0.0209 (6)	
H9	0.5717	0.3976	0.2953	0.025*	
C10	0.46972 (19)	0.5221 (3)	0.3305 (3)	0.0204 (6)	
C11	0.39833 (19)	0.4445 (3)	0.2826 (3)	0.0230 (6)	
H11	0.4009	0.3569	0.2438	0.028*	
C12	0.43633 (18)	0.6451 (3)	0.3827 (3)	0.0197 (6)	
C13	0.47407 (19)	0.7591 (3)	0.4513 (3)	0.0217 (6)	
H13	0.5357	0.7694	0.4680	0.026*	
C14	0.42007 (19)	0.8557 (3)	0.4939 (3)	0.0255 (7)	
H14	0.4452	0.9328	0.5412	0.031*	
C15	0.3294 (2)	0.8434 (3)	0.4694 (3)	0.0285 (7)	
H15	0.2941	0.9122	0.4997	0.034*	
C16	0.2901 (2)	0.7323 (3)	0.4016 (3)	0.0284 (7)	
H16	0.2284	0.7238	0.3842	0.034*	
C17	0.34423 (19)	0.6341 (3)	0.3602 (3)	0.0222 (6)	

### Atomic displacement parameters (Å^2^)

**Table:**

	*U*^11^	*U*^22^	*U*^33^	*U*^12^	*U*^13^	*U*^23^
S1	0.0225 (4)	0.0184 (3)	0.0229 (4)	−0.0009 (3)	0.0070 (3)	−0.0025 (3)
S2	0.0234 (4)	0.0212 (4)	0.0226 (4)	0.0034 (3)	0.0075 (3)	0.0014 (3)
N1	0.0231 (13)	0.0194 (12)	0.0234 (14)	−0.0005 (10)	0.0060 (11)	−0.0047 (10)
N2	0.0197 (12)	0.0236 (13)	0.0211 (14)	−0.0023 (10)	0.0062 (10)	0.0016 (11)
N3	0.0220 (13)	0.0285 (14)	0.0249 (14)	−0.0048 (11)	0.0022 (11)	0.0006 (11)
C1	0.0166 (14)	0.0203 (14)	0.0195 (16)	−0.0007 (11)	0.0027 (12)	0.0006 (12)
C2	0.0223 (15)	0.0239 (15)	0.0181 (16)	−0.0013 (12)	0.0012 (12)	−0.0037 (12)
C3	0.0235 (15)	0.0337 (17)	0.0158 (15)	0.0008 (13)	0.0038 (12)	0.0034 (13)
C4	0.0236 (15)	0.0250 (15)	0.0295 (19)	−0.0006 (13)	0.0045 (13)	0.0106 (14)
C5	0.0262 (16)	0.0198 (15)	0.0277 (18)	0.0005 (12)	0.0041 (13)	−0.0016 (13)
C6	0.0228 (15)	0.0218 (15)	0.0196 (16)	0.0036 (12)	0.0050 (12)	−0.0002 (12)
C7	0.0227 (15)	0.0209 (15)	0.0196 (16)	−0.0006 (12)	0.0032 (12)	−0.0010 (12)
C8	0.0265 (15)	0.0170 (14)	0.0149 (15)	0.0008 (11)	0.0064 (12)	0.0024 (11)
C9	0.0277 (16)	0.0217 (15)	0.0142 (15)	−0.0022 (12)	0.0058 (12)	−0.0003 (12)
C10	0.0268 (15)	0.0201 (14)	0.0140 (15)	−0.0048 (12)	0.0020 (12)	0.0024 (12)
C11	0.0297 (16)	0.0217 (15)	0.0180 (16)	−0.0046 (13)	0.0054 (13)	0.0009 (13)
C12	0.0233 (15)	0.0220 (15)	0.0143 (15)	−0.0039 (12)	0.0044 (12)	0.0040 (12)
C13	0.0227 (15)	0.0248 (15)	0.0177 (16)	−0.0043 (12)	0.0037 (12)	0.0041 (13)
C14	0.0315 (17)	0.0253 (16)	0.0205 (17)	−0.0039 (13)	0.0063 (13)	−0.0028 (13)
C15	0.0291 (17)	0.0296 (17)	0.0277 (18)	0.0022 (14)	0.0077 (14)	−0.0038 (14)
C16	0.0231 (16)	0.0340 (18)	0.0281 (18)	−0.0034 (13)	0.0040 (13)	0.0005 (15)
C17	0.0272 (16)	0.0228 (15)	0.0163 (15)	−0.0057 (12)	0.0025 (12)	0.0034 (12)

### Geometric parameters (Å, °)

**Table:**

S1—C8	1.771 (3)	C5—H5	0.9500
S1—C7	1.816 (3)	C6—H6	0.9500
S2—C8	1.656 (3)	C7—H7A	0.9900
N1—C8	1.337 (4)	C7—H7B	0.9900
N1—N2	1.382 (3)	C9—C10	1.432 (4)
N1—H1	0.8800	C9—H9	0.9500
N2—C9	1.287 (3)	C10—C11	1.373 (4)
N3—C11	1.355 (4)	C10—C12	1.446 (4)
N3—C17	1.384 (4)	C11—H11	0.9500
N3—H3	0.8800	C12—C13	1.402 (4)
C1—C6	1.383 (4)	C12—C17	1.416 (4)
C1—C2	1.407 (4)	C13—C14	1.377 (4)
C1—C7	1.503 (4)	C13—H13	0.9500
C2—C3	1.384 (4)	C14—C15	1.395 (4)
C2—H2	0.9500	C14—H14	0.9500
C3—C4	1.384 (4)	C15—C16	1.385 (4)
C3—H3a	0.9500	C15—H15	0.9500
C4—C5	1.384 (4)	C16—C17	1.384 (4)
C4—H4	0.9500	C16—H16	0.9500
C5—C6	1.383 (4)		
			
C8—S1—C7	102.30 (13)	H7A—C7—H7B	107.6
C8—N1—N2	121.2 (2)	N1—C8—S2	121.3 (2)
C8—N1—H1	119.4	N1—C8—S1	111.7 (2)
N2—N1—H1	119.4	S2—C8—S1	126.97 (17)
C9—N2—N1	115.0 (2)	N2—C9—C10	121.5 (3)
C11—N3—C17	109.3 (2)	N2—C9—H9	119.3
C11—N3—H3	125.4	C10—C9—H9	119.3
C17—N3—H3	125.4	C11—C10—C9	125.3 (3)
C6—C1—C2	118.6 (3)	C11—C10—C12	106.4 (3)
C6—C1—C7	120.1 (3)	C9—C10—C12	128.3 (3)
C2—C1—C7	121.2 (3)	N3—C11—C10	110.4 (3)
C3—C2—C1	120.1 (3)	N3—C11—H11	124.8
C3—C2—H2	119.9	C10—C11—H11	124.8
C1—C2—H2	119.9	C13—C12—C17	118.6 (3)
C4—C3—C2	120.5 (3)	C13—C12—C10	134.9 (3)
C4—C3—H3a	119.8	C17—C12—C10	106.4 (2)
C2—C3—H3a	119.8	C14—C13—C12	118.7 (3)
C3—C4—C5	119.5 (3)	C14—C13—H13	120.7
C3—C4—H4	120.2	C12—C13—H13	120.7
C5—C4—H4	120.2	C13—C14—C15	121.7 (3)
C4—C5—C6	120.3 (3)	C13—C14—H14	119.2
C4—C5—H5	119.9	C15—C14—H14	119.2
C6—C5—H5	119.9	C16—C15—C14	121.0 (3)
C5—C6—C1	121.0 (3)	C16—C15—H15	119.5
C5—C6—H6	119.5	C14—C15—H15	119.5
C1—C6—H6	119.5	C17—C16—C15	117.4 (3)
C1—C7—S1	114.5 (2)	C17—C16—H16	121.3
C1—C7—H7A	108.6	C15—C16—H16	121.3
S1—C7—H7A	108.6	C16—C17—N3	129.9 (3)
C1—C7—H7B	108.6	C16—C17—C12	122.6 (3)
S1—C7—H7B	108.6	N3—C17—C12	107.4 (2)
			
C8—N1—N2—C9	−172.7 (3)	C9—C10—C11—N3	−177.4 (3)
C6—C1—C2—C3	0.8 (4)	C12—C10—C11—N3	1.3 (3)
C7—C1—C2—C3	−177.1 (3)	C11—C10—C12—C13	175.2 (3)
C1—C2—C3—C4	−1.0 (4)	C9—C10—C12—C13	−6.2 (5)
C2—C3—C4—C5	0.5 (4)	C11—C10—C12—C17	−1.0 (3)
C3—C4—C5—C6	0.2 (4)	C9—C10—C12—C17	177.7 (3)
C4—C5—C6—C1	−0.3 (4)	C17—C12—C13—C14	0.0 (4)
C2—C1—C6—C5	−0.2 (4)	C10—C12—C13—C14	−175.8 (3)
C7—C1—C6—C5	177.7 (3)	C12—C13—C14—C15	−0.6 (4)
C6—C1—C7—S1	113.9 (3)	C13—C14—C15—C16	0.4 (5)
C2—C1—C7—S1	−68.2 (3)	C14—C15—C16—C17	0.4 (5)
C8—S1—C7—C1	103.7 (2)	C15—C16—C17—N3	175.7 (3)
N2—N1—C8—S2	−177.5 (2)	C15—C16—C17—C12	−1.1 (4)
N2—N1—C8—S1	2.6 (3)	C11—N3—C17—C16	−176.7 (3)
C7—S1—C8—N1	179.0 (2)	C11—N3—C17—C12	0.4 (3)
C7—S1—C8—S2	−0.9 (2)	C13—C12—C17—C16	0.9 (4)
N1—N2—C9—C10	−178.3 (2)	C10—C12—C17—C16	177.8 (3)
N2—C9—C10—C11	172.6 (3)	C13—C12—C17—N3	−176.6 (2)
N2—C9—C10—C12	−5.8 (5)	C10—C12—C17—N3	0.3 (3)
C17—N3—C11—C10	−1.1 (3)		
